# Supplementary material for: The chromatin landscape of the euryarchaeon Haloferax volcanii
Source: Genome Biol. 2023 Nov 6;24:253. doi: 10.1186/s13059-023-03095-5 (PMC10626798; doi:10.1186/s13059-023-03095-5)
Supplement: Supplementary file 1 — Additional file 1: Supplementary Figure 1. TSS enrichment levels in ATAC-seq data for Suflolobus islandicus. Supplementary Figure 2. Correlation between the extent of chromatinization and GC content in Haloferax. Supplementary Figure 3. Coordination between chromatin accessibility and transcriptional activity within H. volcanii operons. Supplementary Figure 4. Differential ATAC-seq in KAS-seq analysis for standing H. volcanii cells. [file 13059_2023_3095_MOESM1_ESM.pdf]

Supplementary Materials

Supplementary Figures

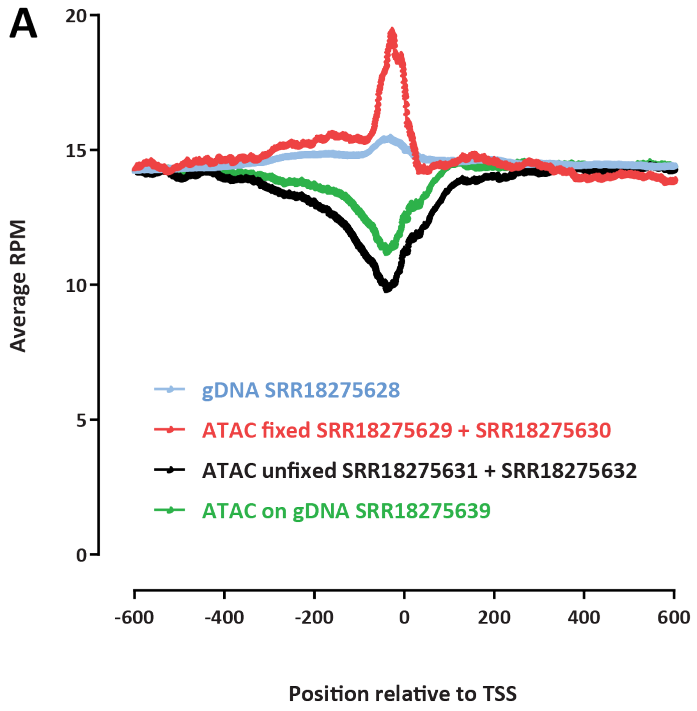

B

Dataset	TSS ratio
gDNA SRR18275628	1.05
ATAC fixed SRR18275629 + SRR18275630	1.11
ATAC unfixed SRR18275631 + SRR18275632	0.80
ATAC on gDNA SRR18275639	0.88

Supplementary Figure 1: TSS enrichment levels in ATAC-seq data for *Suflolobus islandicus*. (A) TSS metaprofiles for ATAC-seq in fixed and unfixed cells and for genomic DNA libraries. (B) TSS scores for each dataset.

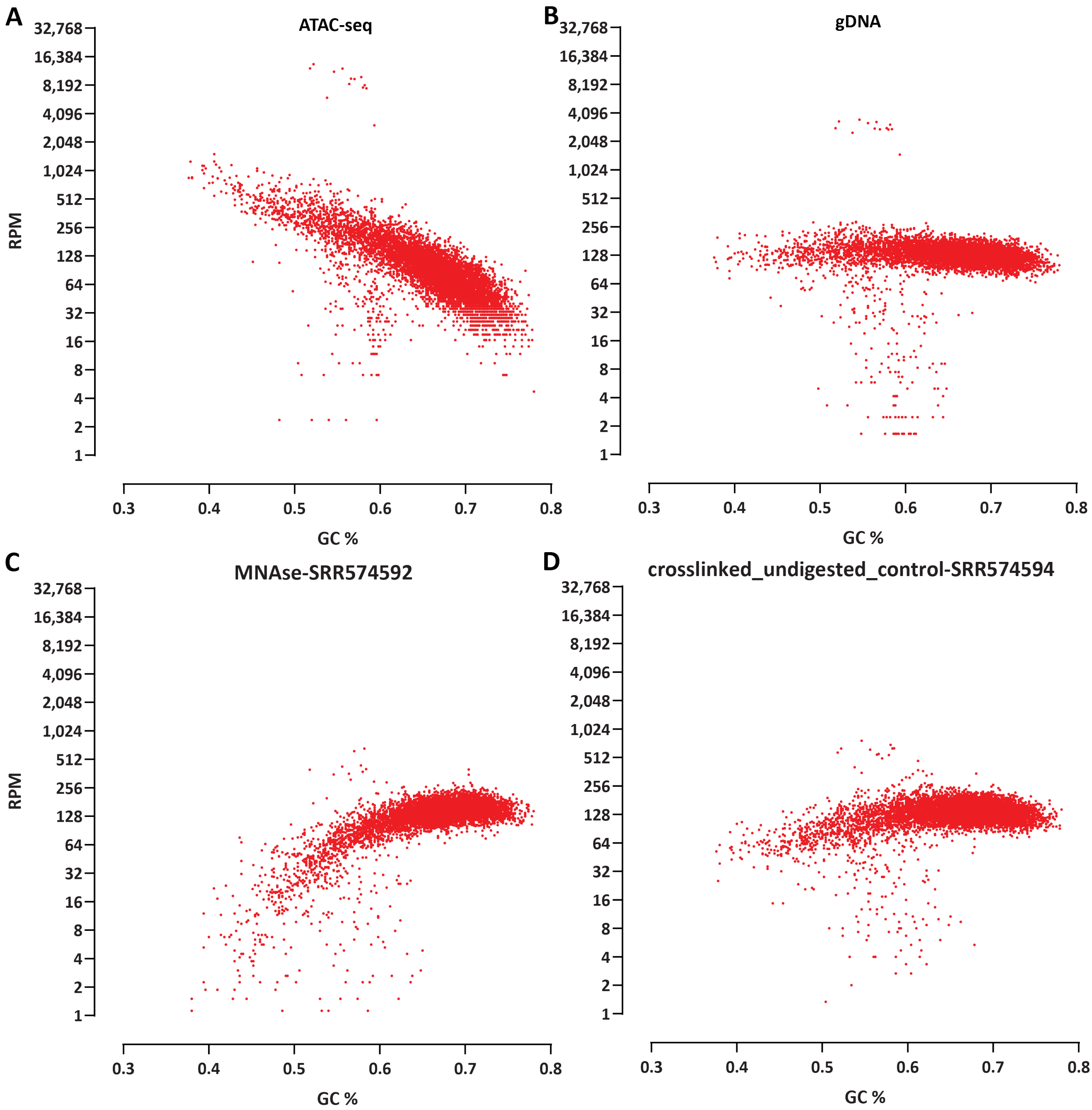

Supplementary Figure 2: Correlation between the extent of chromatinization and GC content in *Haloferax*. The genome was split in 500-bp bins and RPM values and GC% were calculated for each bin. (A) ATAC-seq; (B) Tagmented naked genomic DNA control; (C) MNase-seq (external dataset); (B) Crosslinked undigested control (external dataset).

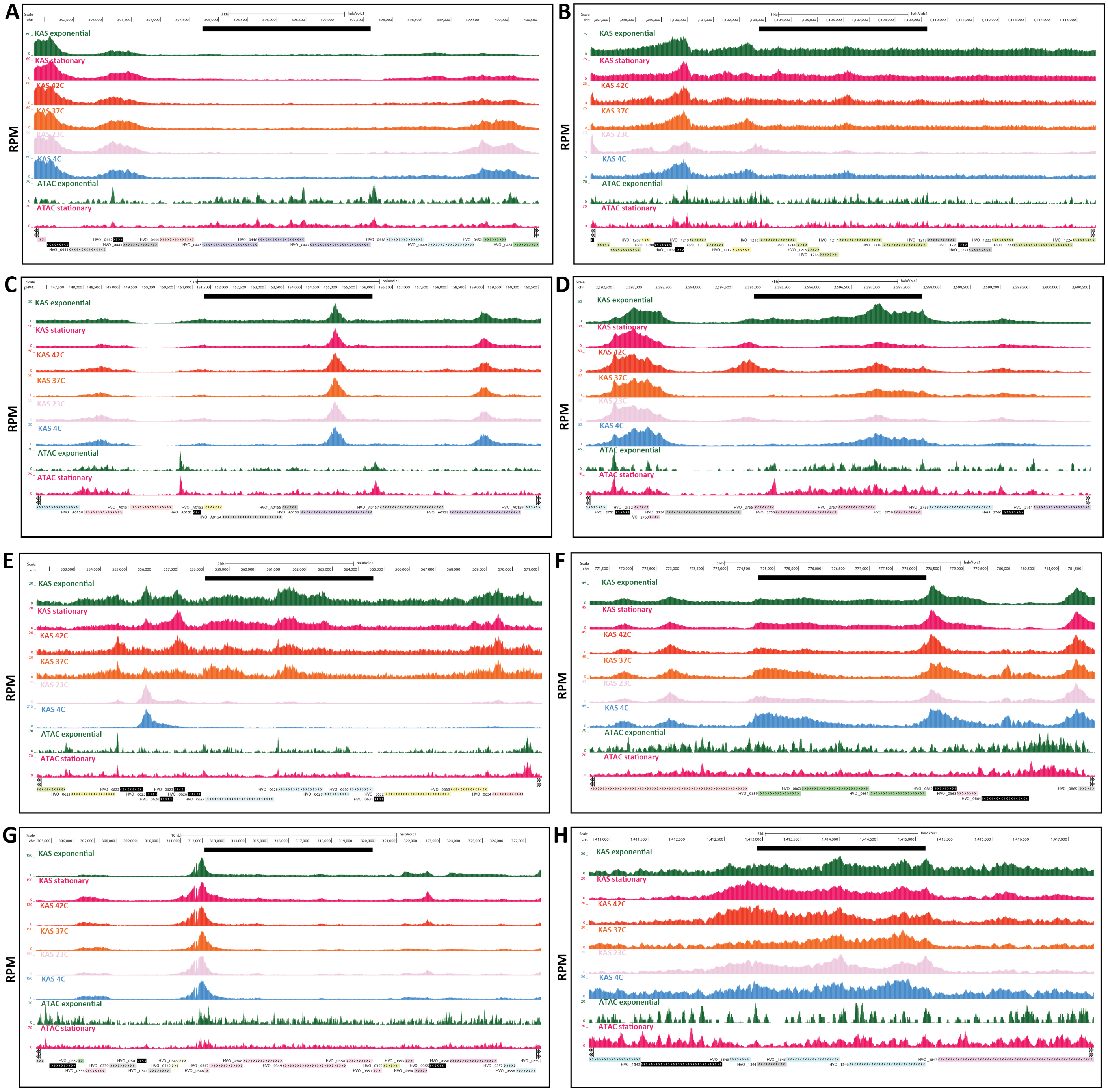

Supplementary Figure 3: Coordination between chromatin accessibility and transcriptional activity within *H. volcanii* operons. Black bar shows the operon boundaries. (A) Putative phosphate-phosphonate ABC transporter. (B) Flagellar cluster (FlaCE, FlaF, FlaG, FlaH, FlaI, FlaJ). (C) Urease accessory protein operon (UreG, UreD, UreE, UreF). (D) 50S ribosomal proteins L12, L10, L1, L11. (E) Putative ABC transporter. (F) FeS assembly genes SufC, SufB, SufD. (G) RNA Polymerase subunits. (H) Dihydroxyacetone kinase subunit L, subunit DhaK, and phosphotransfer subunit.

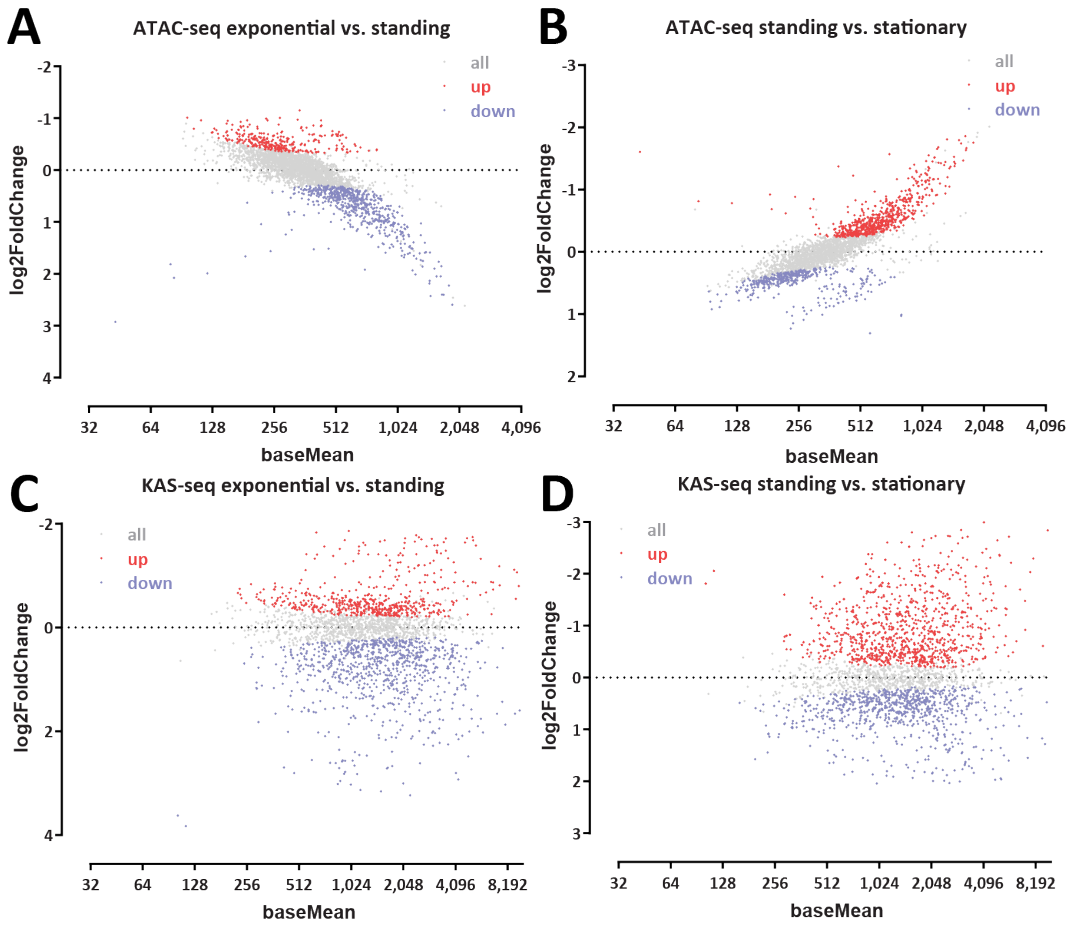

Supplementary Figure 4: Differential ATAC-seq in KAS-seq analysis for standing *H. volcanii* cells. (A-B) Differential chromatin accessibility; (C-D) Differential KAS-seq levels.
