# Supplementary material for: The chromatin landscape of the euryarchaeon Haloferax volcanii
Source: Genome Biol. 2023 Nov 6;24:253. doi: 10.1186/s13059-023-03095-5 (PMC10626798; doi:10.1186/s13059-023-03095-5)
Supplement: Supplementary file 2 — Additional file 2. Peer review history. [file 13059_2023_3095_MOESM2_ESM.docx]

**Review history**

**First round of review**

**Reviewer 1**

This manuscript by Marinov and colleagues deploys a battery of state-of-the-art functional genomics techniques to investigate transcription (and its link to chromatin architecture and DNA accessibility) in the halophilic archaeon Haloferax volcanii. Amongst other things, the authors quantify genome accessibility (using ATAC-Seq and single molecule approaches) alongside ongoing transcription (using KAS-Seq) to demonstrate that - unlike in eukaryotes - changes in transcriptional output do not coincide with changes in chromatin accessibility. Rather, chromatin accessibility is mostly constant across the conditions the authors investigate. This is an important finding because it supports a model where chromatin remodelling is not required for gene activation, suggesting that Haloferax behaves more like bacteria when it comes to their transcriptional ground state (sensu Struhl 1999 Cell 98:1-4), with promoters constitutively accessible even though the global level of chromatinization appears relatively high.

I believe that the results presented here will be an important reference point for future investigations of archaeal transcription and chromatin, especially when it comes to investigating archaea with genuinely (see below) histone-based chromatin and to establish whether chromatin remodeling for transcriptional modulation is a something unique to eukaryotes or also present in archaea.


Major comments:

1. Having read the pre-print as well as the manuscript presented here, I appreciate the additional context that has been added, including discussion of recent findings (notably from Amy Schmid's lab) challenging the notion that histones are a major contributor to chromatin in Haloferax volcanii. These findings have pointed out that initial MNase digestion by Ammar et al (2012 eLife), which seemed to reveal nucleosome-sized protected fragments, could not be repeated, that halophilic histones (hstA, hpyA) do not bind genome-wide but in a limited number of locations (Sakrikar et al 2023 mBio), and that abundance of these histones is far too low to act as a global organizer of chromatin (e.g. Hocher et al 2023 Nat Micro), unlike in other archaea and in eukaryotes. The author's own data is perfectly consistent with this body of evidence: they find no indication for (hyper)nucleosomal complexes in the ATAC-Seq fragment length distribution, no obvious footprints of protection in their single molecule traces, in fact no evidence at all that histones shape accessibility landscape of the H. volcanii genome. Yet the authors seem to lack the conviction of their own data and - perhaps to rescue the original framing of understanding the evolution of histone-based chromatin - argue that nucleosomal complexes are so dynamic that they lack stable positioning and don't leave recognizable footprints and that histones are really the only known entity that could confer restricted accessibility. Both of these arguments are flawed, as I will illustrate further below. The more likely explanation - given the weight of the data here and elsewhere - is that they don't find nucleosome-sized footprints because they do not exist (or are very rare). What the authors describe as "puzzling" (Discussion p.11) is only so because of the felt need to preserve a central role of histones in Haloferax chromatin. I would urge the authors to look at the totality of the data and present their conclusions without a pre-conceived notion about the pre-eminence of histones, which has led them to make some odd arguments, beginning with this:

* The authors suggest that nucleosomes in archaea are dynamic (initially on P.3 line 14), citing some MD/cryoEM results from the Luger lab. The study by Bowerman et al did indeed describe archaeal hypernucleosomes as dynamic but not in the way presented by the authors. Bowerman et al did not propose sliding or dynamic repositioning (p3. Line 33 right hand column). Rather, they argued that larger oligomeric complexes might spontaneously open up, in a way that one would not expect to affect positioning. Adding a fixation step is perfectly reasonable to prevent losing native associations but arguing that this step is necessary because of particularly dynamic nucleosomal complexes is not justified. Similarly, in species where hypernucleosome formation has been found (T. kodakarensis, M. fervidus, M. jannaschii), for which dynamic breathing was proposed (Bowerman et al 2019 eLife) and where histones are abundant (see e.g Hocher et al 2023 Nat Microbiol), you retrieve perfectly well-positioned footprints, so evoking dynamism as the driver behind the lack of footprints makes no sense here.

* Relating to the point that "No protein other than histones…" (p. 12) is known to confer absolute protection/occupancy, this is not quite right. For example, we previously found peaks of MNase resistance at/around promoters in the histone-free organism Thermoplasma acidophilum (Hocher et al 2019 eLife) so proteins other than histones can, in fact, have histone-like properties. The data presented here and elsewhere suggests that protection in Haloferax is very unlikely to come from histones and more likely to be from an as yet uncharacterized protein or set of proteins.

2. The authors infer a similar (slightly lower) absolute accessibility of the H. volcanii compared to the yeast genome. I think readers would benefit from a more in-depth discussion (including caveats where necessary) of how comparable these data really are. Are the cross-linking protocols used here and previously identical? If not, how would this affect our ability to compare absolute accessibility. Does cross-linking itself perhaps hide key differences in the nature of chromatin in yeast and Haloferax? For example, for the sake of argument, let's say that chromatin is more dynamic (higher turnover of components, greater mobility of proteins along DNA etc.) in H. volcanii. Would greater accessibility that comes with this dynamism be obscured by fixation? Have similar experiments been done in bacteria? If so, can these be used to establish an accessibility baseline for histone-free systems?

3. The authors present one of the first single-molecule investigations of archaeal chromatin. As far as I can tell from the population of traces in Figure 2, there is no evidence for alternative chromatin states across chromosomal copies. This is interesting in the context of understanding how genomes of polyploid organisms (such as H. volcanii) are organized. It has remained unclear, for example, whether some chromosomal copies in a given cell might be transcriptionally inactive and simply used as back-up copies. The authors' data seems to suggest that this is not the case (or at least that such inactivity is not reflected in differential chromatin state). I think it would be worth commenting on how their data (and approach) can critically inform these questions.

4. The authors make a qualitative statement that the accessibility landscape in yeast and Haloferax looks rather similar. I would have liked to see this investigated a bit more quantitatively. In particular, I was surprised that the authors did not train at least a minimal model (for Haloferax and yeast) to explore the extent to which differential accessibility can be predicted by sequence alone. Is sequence a stronger predictor in Haloferax? Does addition of MNase data help predict accessibility in yeast and Haloferax to the same extent?

Minor comments:

* Labels for Fig 1 panels C and D are so small as to be illegible. These panels should be resized.
* Figure 1I. I find the magnification factor representation confusing and it's impossible to see anything for the smaller plasmids. Could they all be made the same size and relative scale indicated independently?
* P.3 line 22: If you want to be comprehensive about it, MNase-Seq was also done more recently for M. fervidus (Rojec et al 2019 eLife), T. acidophilum (Hocher et al 2019 eLife), and M. jannaschii (https://europepmc.org/article/ppr/ppr595633).
* P.3 line 33: HpyA is the name of the Halobacterium salinarum histone; the H. volcanii histone is HstA
* P.3 line 51 rhs: I struggle to see a 60-70bp periodicity in Fig 1B. If the authors want to claim one, they should show this more formally, e.g. using a Fourier transform analysis.
* What is happening on the main chromosome at 10 o'clock in Figure 4A. There is a broad region of elevated signal. What is it?
* P8. Regarding limited prior study of transcription inside archaeal operons, it should be noted that there is some recent work in this area, including on H. volcanii (https://www.biorxiv.org/content/10.1101/2019.12.18.880849v2.full)
* P11. Line 53: "… display a chromatin organization very similar to that of eukaryotes". This is misleading. It would be much more appropriate to say "display an accessibility landscape similar to…"
* The authors suggest an experiment of expressing a halophilic histone in yeast. This has an approximately 0% of succeeding given the different pI of halophilic histones. Other archaeal histones, yes; halophilic ones, no.

Typographical amendments:

* Abstract:
o Remove "the" from "provide a foundation for the future functional studies"
* Introduction:
o P.2 Line 50: capitalize "Earth"
o P.3 line 19: Is there a word missing here? Something like "scant" or "limited" before "direct experimental investigation"?
o P3. Line 49: should be "promoter regions" not "promoters regions"
o P3. Line 54: word missing here. "occupancy levels over the H. volcanii […]" TSS?
* Results
o P8. Line 13 rhs: remove "but" from "conditions we assessed, but…"
o P. 10 line 50: should be "In stark contrast to the lack of …."


It is my policy to sign my reviews.

Tobias Warnecke

**Reviewer 2**

Archaea are the progenitors of eukaryotes and undoubtedly supplied the original histone proteins that dominate all eukaryotic chromatin today. While most archaea encode histones, their expression has been demonstrated to be radically different in select clades and thus the extent to which histones influence archaeal genome organization and archaeal gene regulation is likewise radically different. While some archaeal clades express histone proteins at sufficient levels to saturate their genomes and thus more closely mimic eukaryotic chromatin, the halophilic archaea do not. All available literature, perhaps best epitomized by the work from the Schmid lab at Duke, suggest that histone levels in halophiles are very low and that rather than histones acting as general DNA binding proteins, that histones in halophilic archaeal species function more like site specific transcription factors.

The current manuscript has major concerns. Most notably, the choice of organism suggests that any results obtained that are statistically supported will not reflect the histone-abundant landscape of many archaeal clades and will rather report the mixed results of minor histone abundance in compilation with many additional nucleoid-binding proteins. The complete absence of any efforts to define what percentage of the genome accessibility is influenced by histones versus other chromatin proteins is a major disappointment and in of itself, precludes any significant report that can be made from the results obtained.

Major concerns:
1. The Authors repeatedly claim accessibility does not correlated with transcription levels but never offer support for such a profound statement in their analyses. Figure 2A-C, absolute occupancy levels surrounding TSS reach ~85%, whereas at TSS, accessibility falls to ~81%. Given the known error in any measurements of this type, can the authors detail what data were (perhaps selectively) used to support this major claim?


2. It would be interesting to see this data faceted by expression levels. That is, what do TSS occupancy levels look like for genes that are expressed in the top 25th percentile vs the bottom 25th percentile? It would be interesting to see if TSS of highly expressed genes have different occupancy levels compared to silenced genes within the same growth conditions. Figure 5 partially addresses this question, but room for quantitative improvements in large. Figure 6 claims to address this, but how comparing transcription and accessibility between growth phases answers this question remains unexplained. Comparisons should be done within the same data set, not between.

3. Page 5, line 3-5 (Fig 1G), results in significant confusion. Fragment length usually refers to the length of a sequenced read pair, which is an artifact of the sequencing protocol. The fragment size distribution in 1G looks as expected for paired end-sequencing, which would not normally be biologically meaningful. Perhaps "fragment" in this context is instead referring to the size of each contiguous accessible region, and not the actual length of each sequenced fragment. Or perhaps the library preparation protocol was modified in some way to specifically address this question. Clarifications would assist with substantiating conclusions.

4. The ATAC-seq method is predictive of accessible DNA regions. The research assumes that inaccessibility is due to chromatinization/histone occupancy, but other proteins are likely binding to the DNA making it non-accessible. Can The authors explicitly make a case for the accessibility assays predominantly measuring histone-based accessibility? It would be more convincing to see a pattern of histone occupancy (like what Fig 1G is likely included to show). Without significant efforts to address this point, essentially all conclusions are not valid.

5. To increase confidence in the methodology, additional details of the sequencing platform will be necessary.

6. Many figures are presented, even when digitally zoomed to the max, at resolutions too small for review. Fig 2D, 5, and 6 are the most concerning.

7. For KAS-seq data, was any correlation noted with GC content?

8. Minor typos throughout.

9. All analysis seem to be done in a whole-genome context with some exceptions made for extrachromosomal plasmids. Is accessibility/single-strandedness enriched anywhere in the genome aside from CRISPR loci (i.e. Gene ontology, viral regions, etc). Any comments on TSSs that appear to be completely inaccessible? Why are transcription bubbles considered but not replication forks that contain ssDNA in much greater abundances??

10. The manuscript contains a massive amount of analysis, but with the exception of Fig 1B, very little can be applied to biological implications.

**Authors’ response to reviewers**

## Reviewer 1:

This manuscript by Marinov and colleagues deploys a battery of state-of-the-art functional genomics techniques to investigate transcription (and its link to chromatin architecture and DNA accessibility) in the halophilic archaeon Haloferax volcanii. Amongst other things, the authors quantify genome accessibility (using ATAC-Seq and single molecule approaches) alongside ongoing transcription (using KAS-Seq) to demonstrate that – unlike in eukaryotes – changes in transcriptional output do not coincide with changes in chromatin accessibility. Rather, chromatin accessibility is mostly constant across the conditions the authors investigate. This is an important finding because it supports a model where chromatin remodelling is not required for gene activation, suggesting that Haloferax behaves more like bacteria when it comes to their transcriptional ground state (sensu Struhl 1999 Cell 98:1-4), with promoters constitutively accessible even though the global level of chromatinization appears relatively high.

I believe that the results presented here will be an important reference point for future investigations of archaeal transcription and chromatin, especially when it comes to investigating archaea with genuinely (see below) histone-based chromatin and to establish whether chromatin remodeling for transcriptional modulation is a something unique to eukaryotes or also present in archaea.

We thank the reviewer for his thoughtful and thorough comments and suggestions.

Major comments: 1. Having read the pre-print as well as the manuscript presented here, I appreciate the additional context that has been added, including discussion of recent findings (notably from Amy Schmid’s lab) challenging the notion that histones are a major contributor to chromatin in Haloferax volcanii. These findings have pointed out that initial MNase digestion by Ammar et al (2012 eLife), which seemed to reveal nucleosome-sized protected fragments, could not be repeated, that halophilic histones (hstA, hpyA) do not bind genome-wide but in a limited number of locations (Sakrikar et al 2023 mBio), and that abundance of these histones is far too low to act as a global organizer of chromatin (e.g. Hocher et al 2023 Nat Micro), unlike in other archaea and in eukaryotes. The author’s own data is perfectly consistent with this body of evidence: they find no indication for (hyper)nucleosomal complexes in the ATAC-Seq fragment length distribution, no obvious footprints of protection in their single molecule traces, in fact no evidence at all that histones shape accessibility landscape of the H. volcanii genome. Yet the authors seem to lack the conviction of their own data and - perhaps to rescue the original framing of understanding the evolution of histone-based chromatin - argue that nucleosomal complexes are so dynamic that they lack stable positioning and don’t leave recognizable footprints and that histones are really the only known entity that could confer restricted accessibility. Both of these arguments are flawed, as I will illustrate further below. The more likely explanation - given the weight of the data here and elsewhere - is that they don’t find nucleosome-sized footprints because they do not exist (or are very rare). What the authors describe as ”puzzling” (Discussion p.11) is only so because of the felt need to preserve a central role of histones in Haloferax chromatin. I would urge the authors to look at the totality of the data and present their conclusions without a pre-conceived notion about the pre-eminence of histones, which has led them to make some odd arguments, beginning with this:

We do not disagree with the reviewer about the current understanding of the nature of chromatin accessibil- ity/protection in *Haloferax*. We have rewritten the relevant portions of the text to even better reflect that.

- The authors suggest that nucleosomes in archaea are dynamic (initially on P.3 line 14), citing some MD/cryoEM results from the Luger lab. The study by Bowerman et al did indeed describe archaeal hypernucleosomes as dynamic but not in the way presented by the authors. Bowerman et al did not propose sliding or dynamic repositioning (p3. Line 33 right hand column). Rather, they argued that larger oligomeric complexes might spontaneously open up, in a way that one would not expect to

affect positioning. Adding a fixation step is perfectly reasonable to prevent losing native associations but arguing that this step is necessary because of particularly dynamic nucleosomal complexes is not justified. Similarly, in species where hypernucleosome formation has been found (T. kodakarensis, M. fervidus, M. jannaschii), for which dynamic breathing was proposed (Bowerman et al 2019 eLife) and where histones are abundant (see e.g Hocher et al 2023 Nat Microbiol), you retrieve perfectly well- positioned footprints, so evoking dynamism as the driver behind the lack of footprints makes no sense here.

We thank the reviewer for these suggestions and have rewritten the text accordingly.

- Relating to the point that ”No protein other than histones. . . ” (p. 12) is known to confer absolute protection/occupancy, this is not quite right. For example, we previously found peaks of MNase resis- tance at/around promoters in the histone-free organism Thermoplasma acidophilum (Hocher et al 2019 eLife) so proteins other than histones can, in fact, have histone-like properties. The data presented here and elsewhere suggests that protection in Haloferax is very unlikely to come from histones and more likely to be from an as yet uncharacterized protein or set of proteins.

We again thank the reviewer for the suggestion and have rewritten the text accordingly while also referencing the *Thermoplasma* study. Technically, the statement quoted is still true as we do not know which protein might be responsible, something that we hope to figure out eventually.

2. The authors infer a similar (slightly lower) absolute accessibility of the H. volcanii compared to the yeast genome. I think readers would benefit from a more in-depth discussion (including caveats where necessary) of how comparable these data really are. Are the cross-linking protocols used here and previously identical? If not, how would this affect our ability to compare absolute accessibility. Does cross-linking itself perhaps hide key differences in the nature of chromatin in yeast and Haloferax? For example, for the sake of argument, let’s say that chromatin is more dynamic (higher turnover of components, greater mobility of proteins along DNA etc.) in H. volcanii. Would greater accessibility that comes with this dynamism be obscured by fixation? Have similar experiments been done in bacteria? If so, can these be used to establish an accessibility baseline for histone-free systems?

We appreciate the reviewer’s concerns and have further clarified these issues in the text.

Briefly, we recover largely identical results at the level of metaprofiles in yeast with and without crosslinking, and the protocol used for yeast and *Haloferax* is indeed identical (we always use the same protocol for bulk fixation except in certain special circumstances that do not apply here).

Fixation is expected to “freeze” things in place, and thus to provide the ability to measure the statistical distribution of accessibility at any given moment. If there is a highly dynamic association of proteins with DNA, that can result in degradation of both ATAC and SMF signal. The reason is that neither treatment is instantaneous – chromatin is exposed to Tn5/MTase for 15–30 minutes, and if DNA is protected for a period much shorter than that, then exposed for a significant amount of time, then protected again, that gives the enzymes the opportunity to cleave/insert/methylate. It is true that such a state can be seen as more “open” than chromatinized eukaryotic DNA outside active regulatory elements, which is protected pretty much permanently.

There has been one bacterial ATAC-seq study that we are aware of (see *bioRxiv* 2021.01.09.426053), and we have internal unpublished data for other species, but no single-molecule footprinting has been carried out on any prokaryote. Thus there is unfortunately nothing to compare to at the moment.

3. The authors present one of the first single-molecule investigations of archaeal chromatin. As far as I can tell from the population of traces in Figure 2, there is no evidence for alternative chromatin states across chromosomal copies. This is interesting in the context of understanding how genomes of polyploid organisms (such as H. volcanii) are organized. It has remained unclear, for example, whether some chromosomal copies in a given cell might be transcriptionally inactive and simply used as back-up copies. The authors’ data seems to suggest that this is not the case (or at least that such inactivity is not reflected in differential chromatin state). I think it would be worth commenting on how their data (and approach) can critically inform these questions.

We agree with the reviewer that this is an important point to explicitly discuss, and have expanded upon it in the revised manuscript.

4. The authors make a qualitative statement that the accessibility landscape in yeast and Haloferax looks rather similar. I would have liked to see this investigated a bit more quantitatively. In particular, I was surprised that the authors did not train at least a minimal model (for Haloferax and yeast) to explore the extent to which differential accessibility can be predicted by sequence alone. Is sequence a stronger predictor in Haloferax? Does addition of MNase data help predict accessibility in yeast and Haloferax to the same extent?

We share the reviewer’s desire for revealing the sequence determinants of chromatin accessibility in *Haloferax*, but we have simply not had much success with obtaining results that we ourselves are confident in and that can be part of an official publication. A big part of the problem is that *Haloferax* has a highly unusual GC content –

*∼* 65% on average, but promoter regions, where accessibility is mostly (but not entirely) concentrated, are more

AT-rich than genes. We have a lot of experience training sequence models in eukaryotes and properly controlling

for enzymatic and sequence biases has emerged as an absolutely critical requirement for extracting meaningful biological conclusions. The nature of the *Haloferax* genome makes that very complicated currently because the best way for controlling biases is to use “negative” regions in the genome where there is some Tn5 cleavage but at low levels, and which otherwise match the accessible regions in terms of sequence composition properties. But with *Haloferax* we don’t have that luxury because of how compact the genome is – in most cases peaks are separated from other peaks only by relatively short stretches of sequence and elevated accessibility does “bleed through” into gene bodies. So we are very afraid that whatever sequence determinants we come up with will be the result of various biases and sequence composition irregularities. In fact, we are afraid that previously published results trying to relate sequence to MNase signal might well be substantially affected by such issues, and we would rather not contribute further confusion to the literature for the time being. We prefer to address this question separately over a larger diversity of species after a deep investigation into the nature of the various biases and how to best control them in the context of highly compact prokaryote genomes.

Minor comments: * Labels for Fig 1 panels C and D are so small as to be illegible. These panels should be resized.

We have resized and rearranged the panels.

- Figure 1I. I find the magnification factor representation confusing and it’s impossible to see anything for the smaller plasmids. Could they all be made the same size and relative scale indicated independently?

We have resized and rearranged these panels too, and also the analogous ones for the first KAS-seq figure.

- P.3 line 22: If you want to be comprehensive about it, MNase-Seq was also done more recently for M. fervidus (Rojec et al 2019 eLife), T. acidophilum (Hocher et al 2019 eLife), and M. jannaschii (https://europepmc.org/article/ppr/ppr595633).

We thank the reviewer for the more recent references. We have incorporated those in the revised manuscript.

- P.3 line 33: HpyA is the name of the Halobacterium salinarum histone; the H. volcanii histone is HstA We thank the reviewer for the correction and have revised the text accordingly.
- P.3 line 51 rhs: I struggle to see a 60-70bp periodicity in Fig 1B. If the authors want to claim one, they should show this more formally, e.g. using a Fourier transform analysis.

We have removed this statement from the revised version of the manuscript.

- What is happening on the main chromosome at 10 o’clock in Figure 4A. There is a broad region of elevated signal. What is it?

This region of elevated signal is unique to the long-term standing culture condition and it is most likely not related to strongly increased transcription but to changes in copy number (but just for this region, not for the whole chromosome). We do not have an explanation for what the nature of the mechanisms responsible for this phenomenon is as we have not seen it in other libraries generated subsequently.

- P8. Regarding limited prior study of transcription inside archaeal operons, it should be noted that there is some recent work in this area, including on H. volcanii (h[ttps://www.biorxiv.org/con](http://www.biorxiv.org/content/10.1101/2019.12.18.880849v2.full))ten[t/10.1101/2019.12.18.880849v2.full)](http://www.biorxiv.org/content/10.1101/2019.12.18.880849v2.full))

We have added this reference to the revised manuscript.

- P11. Line 53: ”. . . display a chromatin organization very similar to that of eukaryotes”. This is misleading. It would be much more appropriate to say ”display an accessibility landscape similar to. . . ”

We agree that the suggested phrasing is more proper and have corrected the text.

- The authors suggest an experiment of expressing a halophilic histone in yeast. This has an approx- imately 0% of succeeding given the different pI of halophilic histones. Other archaeal histones, yes; halophilic ones, no.

We agree with the reviewer that such an experiment would have to use other archaeal histones, and we have revised the text accordingly

Typographical amendments:

- Abstract:
- Remove ”the” from ”provide a foundation for the future functional studies” Corrected.
- Introduction: o P.2 Line 50: capitalize ”Earth” Corrected.
- P.3 line 19: Is there a word missing here? Something like ”scant” or ”limited” before ”direct experi- mental investigation”?

There is a missing word indeed (it appears to have been deleted during a revision of the text). It has been corrected.

- P3. Line 49: should be ”promoter regions” not ”promoters regions” Corrected.
- P3. Line 54: word missing here. ”occupancy levels over the H. volcanii [. . . ]” TSS? Corrected.
- Results o P8. Line 13 rhs: remove ”but” from ”conditions we assessed, but. . . ” Corrected.
- P. 10 line 50: should be ”In stark contrast to the lack of	”

Corrected.

## Reviewer 2:

Archaea are the progenitors of eukaryotes and undoubtedly supplied the original histone proteins that dominate all eukaryotic chromatin today. While most archaea encode histones, their expression has been demonstrated to be radically different in select clades and thus the extent to which histones influence archaeal genome organization and archaeal gene regulation is likewise radically different. While some archaeal clades express histone proteins at sufficient levels to saturate their genomes and thus more closely mimic eukaryotic chromatin, the halophilic archaea do not. All available literature, perhaps best epitomized by the work from the Schmid lab at Duke, suggest that histone levels in halophiles are very low and that rather than histones acting as general DNA binding proteins, that histones in halophilic archaeal species function more like site specific transcription factors.

We thank the reviewer for the comments and suggestions

The current manuscript has major concerns. Most notably, the choice of organism suggests that any results obtained that are statistically supported will not reflect the histone-abundant landscape of many archaeal clades and will rather report the mixed results of minor histone abundance in compilation with many additional nucleoid-binding proteins. The complete absence of any efforts to define what percentage of the genome accessibility is influenced by histones versus other chromatin proteins is a major disappointment and in of itself, precludes any significant report that can be made from the results obtained.

We understand the reviewer’s desire to have the kind of data we present here on archaea as a whole, but the current manuscript is about *Haloferax* specifically.

It is a longer-term goal of ours to have comprehensive data on a wide sampling of archaeal species, but we had to start our studies of archaeal chromatin with one of the readily accessible experimentally systems, and that means *Haloferax* (this study) and *Sulfolobus*.

We do not claim to be making conclusions that cover the whole of archaea in this current manuscript, and have rewritten the text to avoid any such allusions, to the best of our abilities.

Major concerns: 1. The Authors repeatedly claim accessibility does not correlated with transcription levels but never offer support for such a profound statement in their analyses. Figure 2A-C, absolute

occupancy levels surrounding TSS reach *∼*85%, whereas at TSS, accessibility falls to *∼*81%. Given

the known error in any measurements of this type, can the authors detail what data were (perhaps

selectively) used to support this major claim?

We are not sure why the reviewer claims we do not provide support for the statement that accessibility does not correlate with transcription levels. Figure 6 directly compares accessibility and transcription levels and shows that there is no correlation between them, something that Reviewer #1 also agrees with. We will be happy to provide any additional analysis regarding the relationship between accessibility and active transcription that might be requested, but the lack of general correlation between them is something we have indeed already shown directly.

Regarding absolute accessibility levels, these were measured using single-molecule footprinting, which is based on the preferential methylation of accessible chromatin by methyltransferases, and which is a standard way of measuring absolute occupancy (e.g. see PMID: 31694866). The numbers cited – 85% for baseline accessibility and

81% at TSSs – are the aggregate across all *∼*3,000 annotated genes in the *Haloferax volcanii* genome, generated from very high (*≥*100*×* for 200-bp fragments) coverage of the genome, and consistent across multiple experiments. It is therefore implausible that the observed preferential accessibilIty of TSSs (which is also orthogonally supported

by ATAC-seq data) is artifactual.

2. It would be interesting to see this data faceted by expression levels. That is, what do TSS occupancy levels look like for genes that are expressed in the top 25th percentile vs the bottom 25th percentile? It would be interesting to see if TSS of highly expressed genes have different occupancy levels compared to silenced genes within the same growth conditions. Figure 5 partially addresses this question, but room for quantitative improvements in large. Figure 6 claims to address this, but how comparing transcription and accessibility between growth phases answers this question remains unexplained. Comparisons should be done within the same data set, not between.

Figure 6 compares ATAC and KAS across the same condition (panels C and D in the original manuscript; D and E in the revised version).

We have added the requested analysis by quintiles (Figure 6C in the revised manuscript). It corroborates the direct correlation analysis.

3. Page 5, line 3-5 (Fig 1G), results in significant confusion. Fragment length usually refers to the length of a sequenced read pair, which is an artifact of the sequencing protocol. The fragment size distribution in 1G looks as expected for paired end-sequencing, which would not normally be biologically meaningful. Perhaps ”fragment” in this context is instead referring to the size of each contiguous accessible region, and not the actual length of each sequenced fragment. Or perhaps the library preparation protocol was modified in some way to specifically address this question. Clarifications would assist with substantiating conclusions.

“Fragment” is a standard term in the context of ATAC-seq (and other sequencing-based functional genomic assays) and refers to the library fragment distribution, which in the case of ATAC-seq and eukaryotes shows a nucleosomal signature, as shown in the plot below:

This is in fact highly biologically meaningful – in the figure above one observes:

1. Subnucleosomal fragments (the peak around 60-70bp). These fragments derive from two physically close transposition events inside a promoter or an enhancer region),
2. Mononucleosomal fragments (peak around 200-250bp). These fragments derive from two transposition events that encompass a nucleosome; most often this is the flanking nucleosome of a promoter or an enhancer region,

which is why the length is longer than the *∼*147 bp that a nucleosome physically protects.

3. A fainter dinucleosomal peak.

The absence of such a signature in *Haloferax* is in fact significant – we do not expect it to show the same length because archaeal histones (if we assume histones are the key packaging protein) or other proteins are not expected

to protect the exact same *∼*147 bp of DNA that eukaryotic nucleosomes do, but maybe about half of that. We see

no ladder of sub-, mono-, di-, etc. particles, which means that there are no strings of such proteins closely packed

together and in sufficiently large abundance to generate such a signature.

4. The ATAC-seq method is predictive of accessible DNA regions. The research assumes that inac- cessibility is due to chromatinization/histone occupancy, but other proteins are likely binding to the DNA making it non-accessible. Can The authors explicitly make a case for the accessibility assays predominantly measuring histone-based accessibility? It would be more convincing to see a pattern of histone occupancy (like what Fig 1G is likely included to show). Without significant efforts to address this point, essentially all conclusions are not valid.

We make no hard claims about the nature of the proteins conferring protection of DNA from Tn5 insertion and methylation outside of TSSs. We agree that the literature specifically on *Haloferax* points to histones not being sufficiently abundant to account for the observed patterns, but beyond that we cannot say much. We have further rewritten the text to make that more clear.

5. To increase confidence in the methodology, additional details of the sequencing platform will be necessary.

We are not sure what details about the sequencing platform the reviewer refers to.

In terms of library preparation, the protocols used are described in the Methods section at a level of detail that should enable exact reproduction of the experiments.

Regarding how sequenced was done, we carried out standard Illumina NextSeq (for ATAC and KAS) and NovaSeq (for single-molecule footprinting) sequencing. ATAC-seq and KAS-seq datasets were analyzed as fixed 2

*×* 36mers so the sequencing format does not even matter all that much (i.e. whether it is a 75-cycle NextSeq kit or

a 300-cycle NovaSeq). Longer reads are vital for SMF/NOMe-seq. We have added an explicit note in the Methods

section.

Finally, the datasets have been sequenced more than sufficiently deeply as the *Haloferax* genome is very small – only 4,012,900 bp. For the single-molecule footprinting, we achieve 100*×* coverage for fragments of *∼*200 bp length. This would be the equivalent of a couple billion reads for an analogous mammalian experiment. For ATAC-seq

and KAS-seq, we sequenced to a minimum depth of 1M mapped reads for each library (but for some we sequence

as many as 20-30M). The standard practice for mammalian experiment is to generate 30-50M mapped reads per library, but mammalian genomes are nearly 1000*×* larger, i.e. the coverage we have obtained is the equivalent of at minimum a billion, and in some cases more than 10 billion reads where usually only 50M reads are needed.

6. Many figures are presented, even when digitally zoomed to the max, at resolutions too small for review. Fig 2D, 5, and 6 are the most concerning.

We have resized and upgraded figures where possible. Single-molecule tracks are unfortunately difficult to vectorize because the resulting objects become too large to handle.

7. For KAS-seq data, was any correlation noted with GC content?

KAS-seq requires the presence of a G nucleotide in order for ssDNA to be labeled. Whether GC content might have an effect on the efficiency of labeling is therefore a natural concern. However, *Haloferax* exhibits an unusually

high genomic GC content (*∼*65%), thus this is much less of a concern than it is for a species with e.g. 75-80% AT

content.

Indeed, we do not observe positive correlation between KAS-seq signal and GC content, in fact we observe a slight negative relationship:

This likely reflects a combination of lower GC content in promoter regions, which exhibit elevated KAS signal relative to gene bodies, and perhaps lower representation of extremely GC-rich sequences in final sequencing libraries. However, the latter is certainly not the major contributer to the observed effect because naked DNA amplification using very similar PCR settings shows only a very slight negative relationships between GC content and coverage (see Supplementary Figure 2).

8. Minor typos throughout.

We have corrected all typos we could find in the revised manuscript.

9. All analysis seem to be done in a whole-genome context with some exceptions made for extrachro- mosomal plasmids. Is accessibility/single-strandedness enriched anywhere in the genome aside from CRISPR loci (i.e. Gene ontology, viral regions, etc). Any comments on TSSs that appear to be com- pletely inaccessible? Why are transcription bubbles considered but not replication forks that contain ssDNA in much greater abundances??

There is generally no way to distinguish KAS sequences deriving from transcription bubbles from those deriving from replication forks, However, replication forks are much less abundant than transcription bubbles because the frequency of replication events is much lower (orders of magnitude) than that of transcription. They are also not highly localized within a particular gene, and are thus just background noise in typical KAS-seq datasets.

One of the CRISPR loci is unique as the single strongest KAS-seq peak in the genome, but that should by no means be taken to suggest that accessibility and single-strandedness are confined to CRISPR loci. Accessible and highly transcriptionally active regions are abundant all throughout the genome.

Of the *∼*3,000 annotated *Haloferax* proteins, nearly 1,600 are in the “hypothetical protein” category, which

makes it very difficult to analyze functional gene enrichment, and we have refrained from doing so for that reason.

10. The manuscript contains a massive amount of analysis, but with the exception of Fig 1B, very little can be applied to biological implications.

We appreciate the reviewer’s sentiment but will have disagree in this case – our manuscript is the first examination of *Haloferax* chromatin in such detail, and it contains a wealth of general biological insights relating chromatin organization, the transcriptional landscape and the relationship between those in this organism.

If the reviewer is referring to analysis such as what kinds of genes change between conditions, that was simply not what the goals of our study were – what we were interested in are precisely those general properties of chromatin organization mentioned above. The role of assaying different conditions in this context is to provide perturbations that allow assessing the extent of changes in those properties upon shifts in biological states. We have made all the data publicly available for anyone who is interested in the question of which genes exactly change their expression levels upon the shift between exponential and stationary cultures or upon exposure to cold temperatures, but that is simply beyond the scope of our current manuscript, which, as the reviewer also suggests, already contains a huge amount of analysis.

**Second round of review**

**Reviewer 1**

The authors have addressed my comments. Should future revisions be deemed necessary by the editor, the authors might want to consider applying key techniques (notably ATAC-Seq) to an hstA deletion strain (Sakrikar et al mBio 2023). This would nail the contribution of hstA, the previously identified histone, in this organism. Having said that, there are other predicted, very short histone fold proteins in this organism (<60 amino acids), that have yet to be characterized and their existence is not widely known. These have been identified by recent bioinformatics searches (see e.g. https://www.biorxiv.org/content/10.1101/2023.01.26.525422v1, https://www.biorxiv.org/content/10.1101/2023.06.01.543357v2). So perhaps results from an hstA deletion strain wouldn't be as definitive as they appear as far as histones are concerned.

I'm looking forward to seeing similar functional genomic/chromatin characterization from other archaea in the future.

It is my policy to sign my reviews.

Tobias Warnecke

**Reviewer 2**

The revised manuscript is much improved. The manuscript is improved and should be a nice addition to the archaeal chromatin portfolio. I would only ask that the sizing of small figures be increased whenever possible as they remain very difficult to interpret.
